# Eyelid Cutaneous Leishmaniasis: A Case Report

**Published:** 2017-02

**Authors:** Masoud DOROODGAR, Moein DOROODGAR, Abbas DOROODGAR

**Affiliations:** 1. School of Medicine, Shahid Beheshti University of Medical Sciences, Tehran, Iran; 2. Dept. of Medical Parasitology, School of Medicine, Kashan University of Medical Sciences, Kashan, Iran

**Keywords:** Eyelid cutaneous leishmaniasis, *Leishmania major*, Ocular leishmaniasis

## Abstract

Cutaneous leishmaniasis (CL) is the most common parasitic disease transmitted by vectors in Iran. CL is endemic in many urban and rural parts of Iran and usually caused by two species of *Leishmania* as *L. major* and *L. tropica* transmitted to humans from parasite reservoirs by the bite of female sandflies. We report a case of ocular leishmaniasis with eyelid involvement presentation. The patient was a 70-yr-old housewife woman referred to Health Care Center in city of Kashan, central Iran in 2012. The disease was diagnosed by direct smear, culture, and PCR from the lesion. PCR was positive for *L. major.* Her lesion was treated with systemic meglumine antimonate (Glucantime) (20 mg/kg/day) for 20 days.

## Introduction

Leishmaniasis is used to describe the diseases associated with known species of *Leishmania* parasite. The parasites are transmitted by the bite of sandflies (Family: Psychodidae. Subfamily: Phlebotominae). Due to species of the *Leishmania* and the host immune response it can cause different clinical entities (1, 2). Leishmaniasis is endemic in 98 countries with more than 350 million people at risk. CL is the most common form with 1.5 million new cases per year (3). In the old world, common parasites were *L. major* and *L. tropica* transmitted by *Phlebotomus papatasi* and *P. sregenti* respectively. In The new world, CL is caused by *L. Braziliensis*, *L. amazonensis* and *L. mexicana* which vectors are *Lutzomyia wellcomei* and *L. flaviscutellata* (1).

Up to 90% of cases of CL occur in Afghanistan, Algeria, Islamic Republic of Iran, Saudi Arabia and the Syrian Arab Republic, in Bolivia and Brazil, Colombia, Nicaragua and Peru (3). Both form of rural and urban CL, Zoonotic CL (ZCL) and Anthroponotic CL (ACL) are existing in foci of the disease in Iran (4). About 20,000 cases of CL are reported every year and the actual number may be 4 or 5 times higher (4–6). In fact, CL is considered one of the most important parasitic diseases in Iran with a few reports of atypical clinical forms (7).

Annually several cases of CL are reported in endemic area of Kashan (Isfahan Province, central Iran).

**Fig. 1:**
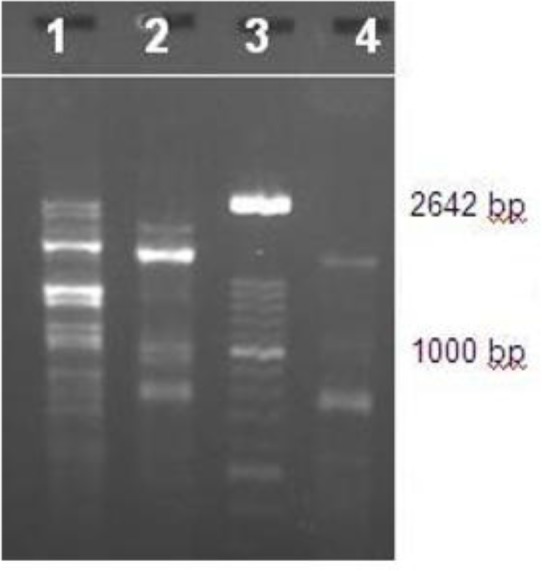
RAPD-PCR technique results in isolated strain and standards: 1- *L. tropica* (MHOM/IR/IR/99), 2- *L. major* (MHOM/IR/75/ER), 3- Marker (100 bp), 4-Unknown strain

Here a case of ocular leishmaniasis (OL) with eyelid involvement is reported. The disease was diagnosed by direct smear, culture of lesion, and PCR for *L. major*, and *L. tropica* by RAPD-PCR method which compared to reference stocks: *L. tropica* (MHOM/IR/IR/99) and *L. major* (MHOM/IR/75/ER) and the results were obtained. The data related to the patient was analyzed by descriptive statistics and bands of PCR product was compared to the standard marker (XIV) strains (Fig. 1).

## Case report

**Fig. 2:**
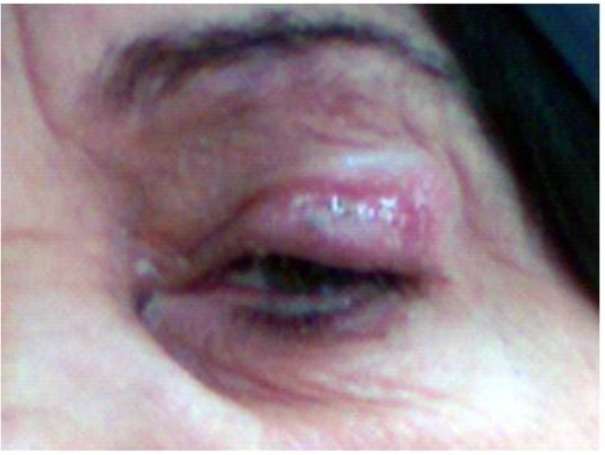
A 70-yr-old woman with lesion of left eyelid

**Fig. 3:**
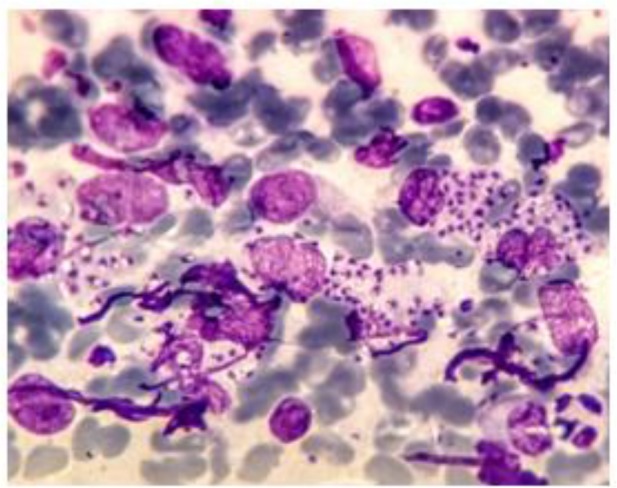
Direct Smear was positive for Leishman bodies (Giemsa stain)

A 70-year-old housewife woman with a complaint of an ulcerative lesion on her upper lid of left eye which was non pruritic was referred to a health center of Kashan (Fig. 2). The lesion size was about 2 cm in 1 cm, was not tender, and was crusty and painless. Her wound was a small nodule initially, enlarged slowly, and after about a month became to current form. At the beginning, the antibiotic treatment was performed for 10 days that failed to heal the lesion. Direct smears were taken from the edge of skin lesion of patient by using vaccine style and were fixed with pure methanol. Following the staining of samples by Giemsa, CL was confirmed by observation of the leishman bodies (amastigotes) by a light microscopy (Fig. 3). After culture examination on NNN medium, PCR was positive for *L. major*.

The patient treated with systemic meglumine antimonate (Glucantime) for 20 days (20 mg/kg/day). Three months after treatment, the lesion healed and only a small scar was remained on the surface of the eyelid.

## Discussion

Cutaneous leishmaniasis is transmitted by the bite of infected female sand flies with *Leishmania* parasites. Because of sandflies, mouthparts are very short; they are unable to bite through clothing. Therefore, CL usually occurs in an uncovered part of body (1, 8). Although the face is an exposed area, but it is an unusual site specially eyelids (6, 9). The possibility of presence of eyelashes and the frequent movements of the eyelids prevent sand fly bites (8, 10). In the season of sandflies activity, more blood sucking is occurred from sunset to sunrise (1). Although bite of eyelids is possible during the rest and sleep, but incidence of cases is much less than bites of the other body parts such as hands and feet. The studies on epidemiology of ocular leishmaniasis confirm the reality that this disease has low incidence (2). In Turkey, 33 ocular leishmaniasis in 987 (1.93%) and 2 out of 718 (0.3%) patients with CL respectively were reported (11, 12). In Iran, Modarres-Zadeh et al. reported four cases of OL from 1950 to 2005 that two of them ended in blindness (2). *L. major* was the main agent of the unusual leishmaniasis in this study and in some cases reported from Iran (6, 10, 13, 14).

Although CL is a self-limiting disease, but untreated ocular leishmaniasis may cause ophthalmologic side effects and can potentially be very serious for eyes. Early diagnosis and vigorous treatment may prevent blindness (2, 15). Now there is no effective vaccine to prevent the disease and treatment is done with drugs (16, 17). The Pentavalent antimonial compounds are the first line drugs of treatment for all forms of leishmaniasis (18).

CL is endemic in many parts of Iran and ocular leishmaniasis should be considered in these areas. In recent years some cases of ocular leishmaniasis has been reported in Iran (2, 9, 15). Diagnosis and treatment of leishmaniasis is done free in Iran. North of Isfahan Province is one the endemic areas for CL caused by *L. major* (19). The described case is a native person who is living in endemic area Kashan County and had no history of trip to other endemic areas of Iran.

## Ethical considerations

Ethical issues (Including plagiarism, informed consent, misconduct, data fabrication and/or falsification, double publication and/or submission, redundancy, etc.) have been completely observed by the authors.
